# Spatial Arrangement Overrules Environmental Factors to Structure Native and Non-Native Assemblages of Synanthropic Harvestmen

**DOI:** 10.1371/journal.pone.0090474

**Published:** 2014-03-04

**Authors:** Christoph Muster, Marc Meyer, Thomas Sattler

**Affiliations:** 1 Zoological Institute and Museum, University of Greifswald, Greifswald, Germany; 2 Musée national d'histoire naturelle Luxembourg, Luxembourg, Luxembourg; 3 Community Ecology, Swiss Federal Institute for Forest, Snow and Landscape Research WSL, Bellinzona, Switzerland; 4 Institute of Experimental Ecology, University of Ulm, Ulm, Germany; 5 Smithsonian Tropical Research Institute, Balboa, Ancon, Republic of Panama; Roehampton University, United Kingdom

## Abstract

Understanding how space affects the occurrence of native and non-native species is essential for inferring processes that shape communities. However, studies considering spatial and environmental variables for the entire community – as well as for the native and non-native assemblages in a single study – are scarce for animals. Harvestmen communities in central Europe have undergone drastic turnovers during the past decades, with several newly immigrated species, and thus provide a unique system to study such questions. We studied the wall-dwelling harvestmen communities from 52 human settlements in Luxembourg and found the assemblages to be largely dominated by non-native species (64% of specimens). Community structure was analysed using Moran's eigenvector maps as spatial variables, and landcover variables at different radii (500 m, 1000 m, 2000 m) in combination with climatic parameters as environmental variables. A surprisingly high portion of pure spatial variation (15.7% of total variance) exceeded the environmental (10.6%) and shared (4%) components of variation, but we found only minor differences between native and non-native assemblages. This could result from the ecological flexibility of both, native and non-native harvestmen that are not restricted to urban habitats but also inhabit surrounding semi-natural landscapes. Nevertheless, urban landcover variables explained more variation in the non-native community, whereas coverage of semi-natural habitats (forests, rivers) at broader radii better explained the native assemblage. This indicates that some urban characteristics apparently facilitate the establishment of non-native species. We found no evidence for competitive replacement of native by invasive species, but a community with novel combination of native and non-native species.

## Introduction

Urban areas are recognized as centres of biological invasions, and there is strong empirical support for a gradient of increasing species richness and abundance of non-native species at the expense of native species towards highly urbanized areas [1]–[5]. Anthropogenic disturbance (e.g., physical disturbance through building, high population and traffic density, pollution, high proportion of impervious surface) is thought to promote the establishment of non-native species by creating resources, reducing the threat of natural enemies and competition, providing physically homogenous environments on a global scale, and supporting passive transport of founders [1], [6], [7]. It is also known that native and non-native species respond differently to socio-economic patterns of settlements, such as gardening practice and individual mobility [8]. However, the issue whether native and non-native species assemblages are shaped by similar processes is a matter of ongoing debate [9], [10]. Some analyses suggest that native communities could be primarily structured by environmental conditions, whereas non-native communities are predominantly structured by human-mediated dispersal [11], [12]. In combination with dispersal limitation, anthropogenic transport may result in autogenic spatial structure of non-native communities which is also referred to as spatially autocorrelated distribution [13], [14]. Since the seminal work by Borcard et al. [15], Legendre [16], and Borcard and Legendre [17] spatial autocorrelation is recognized as an essential component explaining community composition, which is contrast to considering habitat selection as the sole process affecting the occurrence of species in space. Only recently spatial structure was analysed with respect to urban communities [18], [19]. Using Moran's eigenvector maps (MEM) to estimate the spatial component, Sattler et al. [20] found very little spatial variation in urban communities of individual cities of three distinct taxa (spiders, bees, birds). The authors suggested that the absence of spatial structure may be a typical feature of urban species assemblages, owing to the high degree of anthropogenic disturbance. However, the general value of this suggestion needs to be tested with empirical studies from additional regions and taxa.

Synanthropic harvestmen communities in central Europe provide an exceptionally interesting system to study the role of environmental and spatial variation in assemblages of native and non-native species at a regional scale. A relatively high proportion of European harvestmen (Opiliones; app. 100 species in Central Europe [21]) are able to colonize urban habitats [22]. During the last decades, the communities of synanthropic harvestmen in central Europe changed to an unrivalled extent. Up to the 1970s, assemblages of house-dwelling harvestmen were exclusively dominated by the Wall Harvestman *Opilio parietinus*, which is believed to be an archaeozoon that invaded Europe before 1492 from the Near East and Central Asia [23]. Since then, the Mediterranean Red Harvestman *O. canestrinii* has rapidly invaded central and northern Europe, and this neozoon is now by far the most abundant synanthropic harvestmen in large parts of Europe. At the same time, populations of *O. parietinus* declined, and this species is now considered endangered or even extinct in several European countries such as Denmark, the Netherlands, Austria and Germany [24]–[27]. Interspecific competition has been invoked to explain this abundance shift among the congeners, but specific studies remain scarce. However, the changes in harvestmen community in Europe are not restricted to these related species. In recent years, immigration of previously unknown harvestmen species has accelerated with new arrivals being traced in central Europa [28]. In the Netherlands, the number of recorded harvestmen species has increased from 19 in 1963 to 30 in 2009 [26], of which seven were regarded as newcomers since 1993. The situation is similar in Luxembourg, where 19% of the currently known species colonized the country within the last 25 years [29].

In this study, we analysed environmental factors at local and landscape scales as well as spatial variables to explain species distribution and community structure of synanthropic harvestmen in Luxembourg. We pursued the following three objectives: We used variation partitioning among pure spatial, spatially structured environmental, and pure environmental components for the whole community as well as for the assemblages of native and non-native species separately in order to: (1) examine differential effects of landcover variables in structuring the native and non-native fractions of the community; (2) disentangle and compare spatial and environmental components structuring native and non-native assemblages. Furthermore, we (3) determined influential environmental predictors at different spatial scales for individual harvestman species to gain knowledge on species-habitat relationships in this insufficiently studied species group. To contribute to the understanding of the decline of the originally wide-spread Wall Harvestmen *Opilio parietinus*, we interpreted the data to reconsider the hypothesis of its competitive replacement by the invasive congener *O. canestrinii*.

## Materials and Methods

### Ethics statement

Field studies did not involve endangered or protected species and were carried out in accordance with all relevant regulations.

### Study area

Luxembourg is a small central-European country (2.586 km^2^) situated between 49°26′–50°11′N and 5°44′–6°32′E. The country is divided in two natural regions, the Ösling and the Gutland. The Ösling covers the northern third of the area. The elevated plain with deep gorges (300–560 m above sea level) is covered up to 60% by woodland. Mean annual precipitation ranges from 750 to 1000 mm, the mean annual temperature from 7.5 to 9°C. The hilly countryside of the Gutland in the southern two-thirds of the country (150–400 m above sea level) is dominated by agricultural areas, forest coverage is merely 23%. The climate is drier (annual precipitation 700 to 1000 mm) and warmer (mean annual temperature 8.5 to 9.5°C) than in the Ösling [30].

**Figure 1:**
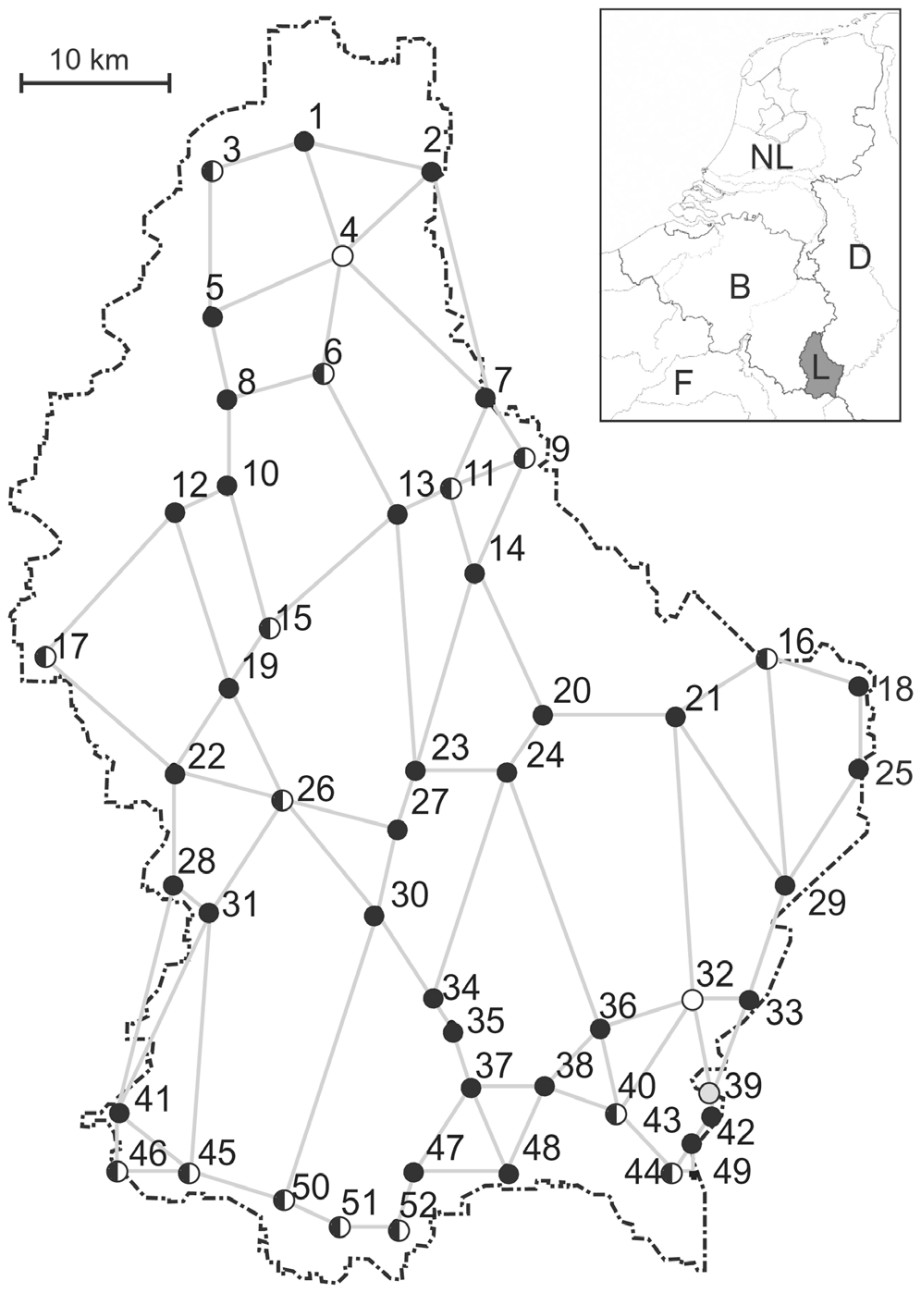
Sampling localities (n = 52) of synanthropic harvestmen in Luxembourg, connected by the Gabriel graph which was used as the definition of the neighbourhood. Numbers indicate settlements and refer to Table S1. Fillings indicate presence/absence of *Opilio canestrinii* and *O. parietinus* (black circles: only *O. canestrinii*, white circles: only *O. parietinus*, half-filled circles: both species, grey circles: none of the two species).

Luxembourg has a high population density (194 people per km^2^). The economic prosperity of the country (rank 3 in the world in terms of gross domestic product per capita) is reflected in the importance of urban areas. The proportion of the urban population is high (85%) and is shared among one large city (Luxembourg City, 20%) and small towns [31]. Synanthropic harvestmen were collected in the Grand Duchy of Luxembourg from 52 human settlements being evenly distributed across the country (Fig. 1). The studied localities include cities, towns and villages and thus cover the full size range of communes (4 localities >10.000 inhabitants, 26 localities between 1000 to 10.000 inhabitants, 22 localities <1000 inhabitants, Table S1). Therefore, we use the term ‘urban’ in a broad sense throughout the paper.

### Field sampling

Abundance data of synanthropic arthropods are difficult to obtain because access to private properties is subject to permissions, and standard methods of arthropod sampling (e. g., pitfall trapping, net sweeping) are of limited use in recording house-dwelling animals. We standardized data collection by sampling in a predefined time unit of 30 min per locality under similar conditions (around noon at warm and dry weather). Starting from a central point in the human settlements, harvestmen were collected by hand by two experienced researchers from vertical structures facing public streets, such as house fronts, walls, gates, monuments etc. at a height from 0 to 2 m. With this method and sufficient experience, wall-inhabiting harvestmen are usually easily detected and almost all central-European synanthropic harvestmen species reach adulthood and their highest densities during the period of our inventory (14 September to 9 October 2009). We therefore assume a high sampling efficiency, but due to the low species and abundance numbers for some localities, we refrained from using rarefaction methods to assess completeness of sampling. Note that different densities of buildings caused the total sampling area in highly urban localities to be smaller than in rural settlements.

### Environmental variables

A thorough inventory of harvestmen in natural and semi-natural habitats in the Grand Duchy of Luxembourg [29] has shown that almost all the species occurring at synanthropic sites actually thrive in or even prefer habitats outside the urban environment. We therefore considered the effects of habitat availability in the surrounding landscape by analysing landcover variables at three different radii (Table 1). We considered landcover variables at radii of 500 m (roughly the sampled area), 1000 m and 2000 m (which is regarded as the maximum walking distance of adult harvestmen) around the centre of the sampling localities. Recent studies of urban arthropods found environmental variables on these radii to be important for explaining variation in communities [18], [20]. A geographical information system was used to extract CORINE landcover (CLC) data for the given areas. The latest vector data from the CLC 2006 census were downloaded from the European Environment Agency (available from http://www.eea.europa.eu/data-and-maps/data/clc-2006-vector-data-version-1). CLC nomenclature is a system of three hierarchical levels [32] from which we derived the environmental variables considered to be useful to analyse synanthropic harvestmen communities. We divided CLC class 1 “Artificial surfaces” into two variables, *Urban* (areas with high percentage of impervious surface, i.e. CLC classes 1.1 to 1.3) and *Parks* (CLC class 1.4). We included *Parks* as a separate predictor variable as we expected an impact of municipal parks on urban biodiversity as shown by recent studies [1], [19]. *Agriculture* and *Forests* were included at CLC level 1 (i.e., all subordinate classes were summed up). These semi-natural habitats (forests are regularly managed in Luxembourg) were included in the study of synanthropic harvestmen because urban habitats may constitute population sinks so that the presence of some species may require continuous immigration from semi-natural source habitats. *Rivers* were included, because running water bodies (CLC category 5.1.1) are known to serve as important migration routes, in particular for non-native species as river banks may be regularly disturbed [1]. Since our study focused on house-dwelling harvestmen, we included the coverage of *Buildings* in the 500 m radius as obtained from a Luxembourg-specific layer as a fine-scale parameter in addition to CLC landcover variables.

**Table 1:** Environmental variables included in the analysis of synanthropic harvestman communities.

		dataset			
variable name	variable description	R500	R1000	R2000	Climate
Urban	CLC 1.1 Urban fabricCLC 1.2 Industrial, commercial and transport unitsCLC 1.3 Mine, dump and construction sites	x	x	x	
Urban∧2	quadratic term of Urban	x	x	x	
Parks	CLC 1.4 Artificial non-agricultural vegetated areas	x	x	x	
Parks∧2	quadratic term of Parks	x	x	x	
Agriculture	CLC 2.1 Arable landCLC 2.2 Permanent cropsCLC 2.3 PasturesCLC 2.4 Heterogenous agricultural areas	x	x	x	
Forest	CLC 3.1 ForestsCLC 3.2 Shrubs and/or herbaceous vegetation associationCLC 3.3 Open spaces with little or no vegetation	x	x	x	
Rivers	Water courses (CLC code 5.1.1.)	x	x	x	
Buildings	Surface area of buildings, derived from WebOBS Luxembourg	x			
Buildings∧2	quadratic term of Buildings	x			
Temp	mean annual temperature				x
Prec	average annual precipitation				x

R500, R1000, R2000 – data at 500 m, 1000 m, and 2000 m radius from starting point of sampling.

The intermediate disturbance hypothesis [33] predicts hump-shaped relationships between disturbance and diversity patterns. As several studies on urban biodiversity have shown such hump-shaped biodiversity patterns along human disturbance gradients [20], [34], we therefore included the predictor variables that are related to human disturbances in settlements (*Urban, Parks* and *Buildings*) as a quadratic term in addition to the linear term.

Moisture and temperature are influential climatic parameters that determine the distribution of harvestmen and other arachnids [35], [36]. We included annual mean temperature and average annual precipitation as climatic parameters in our analyses. Data were obtained from Niedringhaus et al. [30].

### Spatial variables

Different biotic processes such as point introduction with subsequent reproduction and dispersal, interspecific competition, or predation lead to autogenic spatial structure in communities [16], [37]. We used Moran's eigenvector maps (MEM) [37] for detection and quantification of spatial structure in our data with spatial variables. This method is a generalisation of principal coordinates of neighbour matrices (PCNM) [38]. In the original PCNM approach, eigenfunctions are obtained from a truncated pairwise geographic distance matrix between sampling sites. In MEM analysis, more elaborate procedures are available to define neighbourhood, such as Delaunay triangulations, Gabriel graphs or Relative neighbourhood graphs [39]. MEM variables are numerical variables (Eigenvalues) that are built from those eigenvectors of spatially weighted connectivity matrices that maximize the Moran's index of autocorrelation.

The choice of the spatial weighting matrix is the most critical step in spatial analysis [37]. As proposed by these authors, we followed a data-driven approach based on a corrected Akaike information criterion (AIC_C_). Various spatial models, differing in the definition of neighbourhood and weighting functions, were evaluated by their capacity to explain the harvestmen species x site matrix (as measured with adjusted R^2^). We proceeded as follows: 1) Prior to the analysis, the species data were Hellinger transformed and detrended by multiple linear regression on geographic coordinates to remove the effect of a linear gradient [40], [41]. 2) The following types of binary connectivity matrices were considered: Delaunay triangulation, Gabriel graph, Relative neighbour graph and distance criterion, the latter with ten evenly distributed threshold values between 0 and 0.37 (which is half the maximum Euclidean distance among any two localities). Larger distances were not considered, because an increased number of neighbours renders the produced eigenvectors more sensitive to variations in the sampling design [42]. 3) Each neighbourhood matrix was weighted with the following function of distance *f = 1−((d_ij_)^y^/max(d_ij_)^y^)*, where *d_ij_* is the distance of neighbouring sites *i* and *j*. MEM were computed for integers between 1 and 10 for *y* (if *y* = 1 we use a linear weighting function, if y = 10 almost equal weight is given to all connections). For each eigenvector of the best spatial weighting matrix Moran's I was computed and tested in a permutation procedure. Only the significant eigenvectors (p<0.05) of the best spatial model were used in the subsequent variation partitioning. All spatial analyses were run with the R package “spacemakeR” [43].

### Analysis at the community level

We used a hierarchical approach to determine environmental variables that significantly control synanthropic harvestmen communities and which were used in the subsequent variation partitioning. Variation partitioning has proved to be an invaluable method to disentangle the relative contributions of autogenic, spatially structured environmental component, and pure environmental components ([10], [14], [15], [37]; but see [44] for a critique).

Prior to analyses, species data were again Hellinger-transformed and detrended; and landcover data (percentages) were arcsin-transformed following Zar [45].

The first step in data analysis consisted of separate redundancy analyses (RDA) for the variables at each of the three landcover radii, as well as for climatic parameters. For each dataset, we ran forward selection as implemented in the R package “packfor” [46] to identify significant variables (p<0.05, 9999 permutations). To reduce the problems of the forward selection with a simple α-stopping criterion (at e.g., 0.05 level), we followed the two-step procedure of Blanchet et al. [47] in first, including only variables from models for which a global test with all explanatory variables proved to be significant and second, excluding variables that cause the adjusted R^2^ of the current model to exceed the adjusted R^2^ value of the global model. In a second step and to determine the generally important variables, we incorporated the significant variables of each of the four datasets (3 radii + climate) in yet another forward selection. We included the selected environmental variables and the significant MEM variables obtained in the previous step (also forward selection) as spatial variables, into variation partitioning. This analysis was performed with the “varpart” function in the R package “vegan” [48] using the detrended residuals of Hellinger transformed species data as response variables. All analyses were performed for the entire community as well as for sets of native and non-native species separately. Detrended Correspondence Analysis (DCA) of synanthropic harvestmen communities in Luxembourg was performed with the R package “vegan” [48].

### Analysis at the species level

To identify the landcover variables that most strongly influence the distribution of individual species, we applied an information criterion approach. We took the untransformed abundance data of selected species (8 species with >15 total specimens) as dependent variables and the landcover variables listed in Table 1 as explanatory variables. We used the R package glmulti [49] for multi-model inference from general linearized models with quasi-Poisson distribution. For each of the three radii (500 m, 1000 m, 2000 m) all possible candidate models were formulated and analysed (502 models at R500, 120 models each at R1000 and R2000;without considering interactions). An estimate of the importance of the individual variables was computed as the sum of the relative evidence weights of all models in which the term appears. The relative evidence weights of the models were computed as exp(−ΔIC/2), where ΔIC is the difference in the information criterion (IC) between a model and the best model. Evidence weights were normalized so that they sum up to one [50]. This approach is appealing as it takes uncertainty in model-selection into account and at the same time avoids arbitrary cut-off values. The resulting multi-model inference does not depend on a single best model, thus it takes into account the complex relationships in ecological systems [51], [52]. Statistical analyses were performed in R version 2.13.1 [53].

## Results

**Figure 2:**
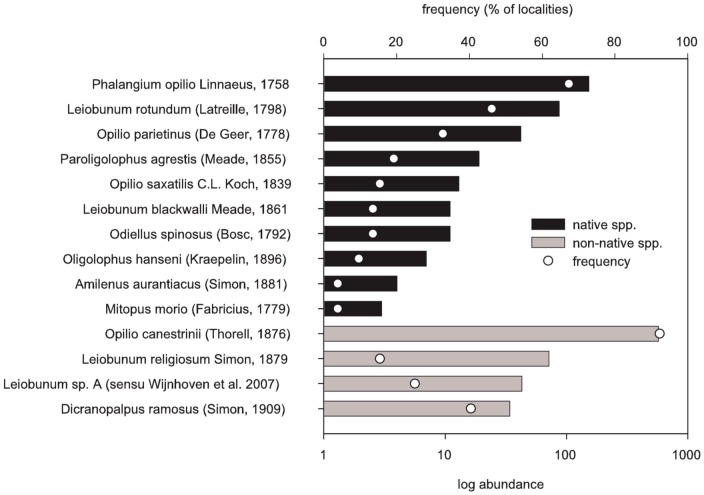
Abundance and frequency of occurrence of synanthropic harvestmen species in Luxembourg.

A total of 1074 harvestmen from 14 species were recorded at the 52 human settlements in Luxembourg (Fig. 2, Table S2). Per locality species numbers range from 1 to 10 (mean 4± SD 1.8), with individuals varying from 1 to 70 (20.7±14.4) per locality. The expansive Mediterranean species *Opilio canestrinii*, which has probably colonized the country since the 1980s (first record in the Netherlands in 1991 [26]), is now by far the most common species dominating communities (53.5% of all specimens) and found at 94% of all localities. On average, 64% (±25%) of the specimens per locality belong to the four non-native species that immigrated within the last 30 years. These are *O. canestrinii, Dicranopalpus ramosus, Leiobunum religiosum* and *L.* sp. A (an unidentified species that has invaded central and western Europe since about the year 2000 [28]). The similarity of synanthropic harvestmen communities in Luxembourg is moderate, with a mean Bray Curtis index of 0.39±0.21 in pairwise comparisons among sites (where 0 means identical communities and 1 stands for entirely different sites that share no species).

### Analysis at the community level

Influential landcover variables were identified in a hierarchical analysis. Significant variables from different scales explained similar amounts of variation (21.9–25.3%, Table 2, top), depending on the radius and the three communities under consideration (all species, native species, non-native species). The native harvestmen fauna appeared more affected by landcover variables at R1000 (24.3%) and R2000 (25.0%), while the non-native assemblage was mostly influenced by conditions on R500 (31.2%) and R2000 (25.7%). On average, the included landcover variables explained slightly more variation in the non-native than in the native species community. Considering the most important environmental variables in the overall analysis (including significant environmental variables of all radii), native species communities were mainly shaped by the availability of natural habitats in the larger radii (forests, rivers), while non-native occurrence were explained by urbanization parameters at small and large radii (Table 2, bottom). The included climatic variables do not exhibit significant effects on the structure of wall-inhabiting harvestmen communities at the regional scale of our study area.

**Table 2:** Environmental factors explaining the composition of synanthropic harvestmen communities and their native and non-native fractions at different spatial scales (radii of 500 m, 1000 m, 2000 m from sample origin) as revealed by the hierarchical analysis.

	All species	Native species	Non-native species
a) R500			
explained variation	24.6%*	n.s.	31.2%**
variables (p<0.05)	Urban∧2		Urban∧2
b) R1000			
explained variation	21.9%*	24.3%**	n.s.
variables (p<0.05)	Forest	Forest, Rivers	
c) R2000			
explained variation	25.3%**	25.0%*	25,7%*
variables (p<0.05)	Parks, Forest, Rivers	Parks, Forest, Rivers	Parks, Urban
d) Climate	n.s.	n.s.	n.s.
e) Total			
explained variation	23.6%**	20.3%*	23.3%*
variables (p<0.05)	Parks (R2000)	Forest (R1000)	Parks (R2000)
	Urban∧2 (R500)	Parks (R2000)	Urban∧2 (R500)
	Forest (R1000)	Rivers (R2000)	
	Rivers (R2000)		

(a–d) Analysis at individual sets of environmental variables (radii, climate). (e) Overall analysis with variables identified in a–d. RDA significance levels: *p<0.05, **p<0.01.

In the selection of a spatial weighting matrix that best fit the data, the lowest AIC_C_ was obtained with the Gabriel graph and *y* = 5 for which we calculated MEM variables. We identified eight spatial MEM variables with significant effects on the community structure of synanthropic harvestmen. The selected MEM variables were associated with spatial patterns at broad (MEM variables 2, 6, 8), medium (14, 21, 22) and fine scales (28, 36).

**Figure 3:**
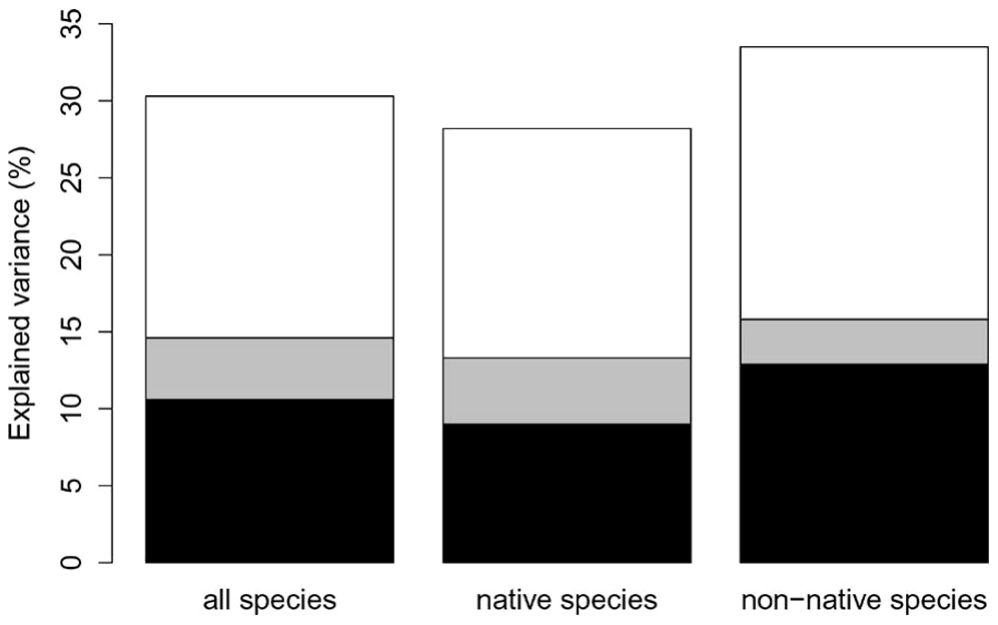
Fractions of purely environmental (black), spatially structured environmental (grey), and purely spatial (white) variation (adjusted R^2^) explaining the composition of synanthropic harvestmen communities and their native and non-native components.

Environmental and spatial variables together explained approximately 30% of the variation in harvestmen community composition (Fig. 3). The proportion of explained variance was somewhat higher in the non-native assemblage (33.5%) as compared to the native fauna (28.2%). The analysis revealed a marked signal of spatial structure, which exceeds environmental control. Pure spatial variation accounted for 54.5% of the explained variation at the community level, this proportion was almost the same in the native (56.2%) and non-native (55.8%) assemblages. Spatially structured environmental variation accounted for a higher relative amount of explained variation (16.2%) in the native species than in the non-native assemblage (9.1%). The relative contribution of pure environmental variation ranged from 34% in the native to 40.7% in the non-native assemblages.

**Figure 4:**
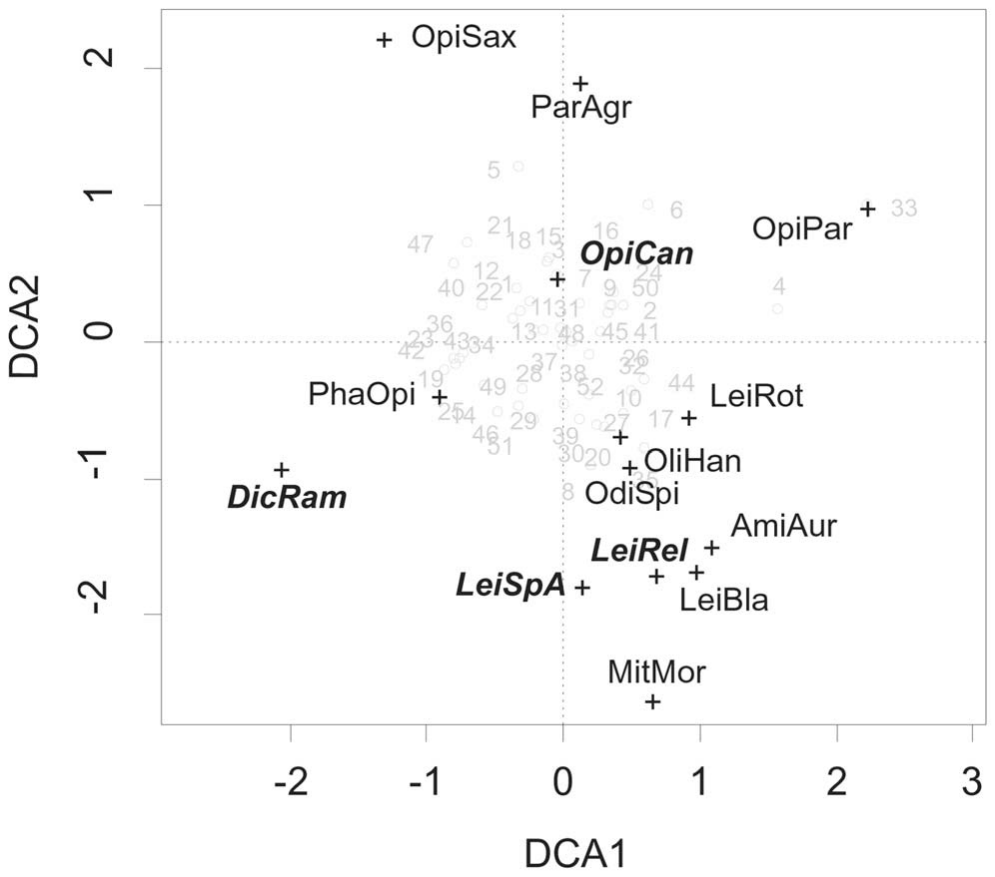
Results of Detrended Correspondence Analysis (DCA) of synanthropic harvestmen communities in Luxembourg illustrating the suggested absence of a displacement effect of native species through non-native species. Native species in normal font, non-native species in bold italics; for species names see Fig. 2; sampling localities are shown in grey (cf. Table S2). Eigenvalues: DCA1 0.284, DCA2 0.258

Detrended Correspondence Analysis (DCA) of the harvestmen communities (Fig. 4) illustrates that the non-native species are mixed within the native species. Comparing environmental vectors with the results of the ordination indicates that DCA axis 1 is positively correlated with forest coverage at all three radii, axis 2 is positively correlated with agricultural areas at all radii. The central position of the non-native *O. canestrinii* demonstrates its ubiquitous distribution without special habitat preferences.

### Analysis at the species level

The sum of the relative evidence weight of all models that include the respective variable provides information about the effect magnitude of single environmental variables at the species level. Species show idiosyncratic relationships to the different land cover variables at the three radii, so no general pattern valid for all harvestmen could be identified. Increased urbanization (variable *Urban*) promoted the abundance of most non-native species (*D. ramosus, L. religiosum, O canestrinii*) but showed less substantial effects on native species (Table 3). But even in the urbanophilic neobiota, high levels of urbanization tend to cause reverse effects, as seen in the negative relationships with the quadratic term (with the exception of *L.* spec. A on R500). This means that populations of these non-native species peak at intermediate levels of urbanization. An increasing fraction of parks at multiple radii positively correlates with occurrence of the non-native *L. religiosum* as well as of *L. rotundum*, its native congener. Increasing fractions of semi-natural land covers such as agricultural areas and forests have a positive influence on the presence of the endangered *O. parietinus*. Rivers appear not to be of substantial relevance in the dispersal process of neither non-native nor native harvestmen, with the exception of *Phalangium opilio*.

**Table 3:** Landcover variables explaining the occurrence of the eight most common synanthropic harvestmen species per radii.

	Buildings	Buildings∧2	Urban	Urban∧2	Parks	Parks∧2	Agricult.	Forest	Rivers
**non-native species**									
*Dicranopalpus ramosus*									
R500	0.65 (−)	0.51 (+)	0.68 (+)	0.48 (−)	0.36 (+)	0.43 (−)	0.24 (+)	0.25 (+)	0.36 (+)
R1000			0.76 (+)	0.37 (−)	0.49 (+)	0.64 (−)	0.28 (+)	0.30 (+)	0.26 (+)
R2000			**0.90 (+)**	0.48 (−)	0.73 (−)	0.29 (−)	0.33 (−)	0.34 (−)	0.23 (−)
*Leiobunum religiosum*									
R500	0.30 (−)	0.30 (+)	0.27 (+)	0.29 (−)	**1.0 (+)**	**1.0 (−)**	0.26 (+)	0.26 (+)	0.24 (−)
R1000			0.28 (+)	0.29 (−)	0.53 (+)	0.61 (+)	0.26 (+)	0.26 (+)	0.26 (−)
R2000			0.35 (+)	0.45 (−)	**0.99 (+)**	0.33 (+)	0.24 (+)	0.25 (+)	0.26 (−)
*Leiobunum* sp. A									
R500	0.26 (−)	0.31 (+)	0.38 (−)	**0.91 (+)**	0.25 (+)	0.25 (−)	0.24 (−)	0.24 (−)	0.24 (−)
R1000			0.51 (−)	0.57 (−)	0.16 (−)	0.25 (+)	0.26 (−)	0.26 (−)	0.25 (−)
R2000			0.53 (+)	0.41 (−)	0.34 (−)	0.37 (+)	0.30 (−)	0.28 (−)	0.27 (−)
*Opilio canestrinii*									
R500	**0.87 (−)**	0.74 (+)	**0.92 (+)**	**0.85 (**−**)**	0.60 (+)	0.59 (−)	0.59 (+)	0.71 (+)	0.30 (−)
R1000			0.54 (+)	0.41 (−)	0.26 (+)	0.26 (−)	0.46 (+)	0.37 (+)	0.61 (−)
R2000			0.59 (+)	0.37 (−)	0.36 (−)	0.27 (+)	0.34 (+)	0.33 (+)	0.75 (−)
**native species**									
*Leiobunum rotundum*									
R500	0.36 (−)	0.28 (+)	0.24 (+)	0.24 (−)	**1.0 (+)**	**1.0 (−)**	0.27 (+)	0.35 (+)	0.22 (+)
R1000			0.33 (+)	0.41 (−)	**1.0 (−)**	**1.0 +)**	0.36 (+)	0.75 (+)	0.22 (−)
R2000			0.31 (+)	0.35 (−)	0.47 (+)	**1.0 (+)**	0.40 (+)	0.73 (+)	0.23 (+)
*Opilio parietinus*									
R500	0.29 (−)	0.36 (+)	0.70 (+)	0.36 (−)	0.24 (−)	0.26 (+)	**0.85 (+)**	**0.91 (+)**	0.25 (−)
R1000			0.49 (+)	0.59 (+)	0.23 (+)	0.23 (−)	**0.84 (+)**	**0.88 (+)**	0.26 (−)
R2000			0.34 (+)	0.29 (+)	0.29 (+)	0.27 (−)	0.29 (+)	0.38 (+)	0.38 (−)
*Paroligolophus agrestis*									
R500	0.25 (+)	0.27 (−)	0.28 (+)	0.28 (−)	0.23 (+)	0.23 (−)	0.47 (+)	0.27 (−)	0.31 (−)
R1000			0.31 (−)	0.31 (−)	0.24 (+)	0.24 (−)	0.28 (−)	0.25 (−)	0.37 (−)
R2000			0.29 (−)	0.27 (−)	0.24 (−)	0.24 (−)	0.28 (−)	0.29 (−)	0.36 (−)
*Phalangium opilio*									
R500	0.27 (+)	0.24 (−)	0.31 (−)	0.27 (+)	0.24 (+)	0.24 (−)	0.48 (+)	0.26 (−)	**0.96 (+)**
R1000			0.39 (−)	0.31 (+)	0.30 (+)	0.30 (−)	0.34 (−)	0.33 (−)	**0.96 (+)**
R2000			**0.81 (−)**	0.26 (−)	0.28 (−)	0.28 (−)	**0.80 (−)**	**0.80 (−)**	0.64 (+)

The values represent the estimated importance of the variables (relative evidence weight) across all candidate models including the respective variables. Values ≥0.8 are shown in bold; the threshold of 0.8 was used to identify important variables [50]. +/− indicates positive/negative relationship with the predictor variable.

## Discussion

Communities of wall-inhabiting harvestmen in Europe are composed of a high proportion of non-native species and thus offer a unique opportunity to study invasion-related processes. In Luxembourg, we found four non-native species to reach an exceptional 64% (SD  =  ±25%) of all specimens per locality. In order to gain insights in processes structuring synanthropic harvestmen communities, we partitioned out the spatial and environmental components explaining variation in the composition. Landcover variables, climatic variables and spatial MEM variables explained some 30% of the total variation (adjusted R^2^). This is more than in many similarly designed studies, but the most striking outcome is the apparent high spatial structure in the wall-inhabiting harvestmen assemblage.

### Strong effects of spatial structure

At the regional scale of this study (minimum of 1 km and maximum of 73 km between sampling locations) we determined that the pure spatial component explained 15.7% of the total variance of the harvestmen community, which is more than 50% of the total explained variance. This points at the fact that biotic processes (e.g. dispersal, interspecific competition, predation) create high autogenic spatial structure in harvestmen communities. Biotic processes seem to outperform processes related to the environment, i.e. habitat selection. The spatial component also explained more of the environmental and mixed component in the native and non-native fractions of the community (56.2% and 55.8% of total explained variance, respectively). Similar high proportions of the pure spatial component, exceeding 15% of the total variance or 50% relative to environmental and mixed effects, have rarely been reported in the literature (but see [54] for cyanobacteria/algae across central European cities; [55] for endemic ground beetles on Madeira). At the local scale of a few kilometres within cities, Sattler et al. [20] found near absence of pure spatial variation in urban spider, bee and bird communities (max. 3.5% of total variance). Our study of synanthropic harvestmen in Luxembourg is an example at the regional scale (10′s of km), resulting in 15.7% of variance explained by pure spatial variation. At a broader scale studying European cities (100′s of km), Chytrý et al. [54] observed pure spatial variation to explain from 4.6% in plants to 21.2% in cyanobacteria/algae.

At first sight our results seem to be at odds with the conclusions of Sattler et al. [20], who stated that the absence of the spatial component to explain urban animal communities might be a special feature of urban assemblages due to continuous human disturbance that destroy or inhibit the development of autogenous spatial structure. The ecology of harvestmen and spatial scale might be keys to understanding this apparent contradiction, an interpretation in line with recent studies that emphasise the scale-dependence of relationships between species assemblages and spatial and environmental variables [55], [56]. As pointed out above, Sattler et al. [20] considered spatial variables at local scale *within* cities, while here we included spatial variables *between* human settlements. While human disturbance as a typical urban process may be partially reflected by a study of spatial variables within cities, the processes explaining spatial variables between towns and settlements are more complex. Synanthropic processes such as disturbance by humans are expected to be only one of many processes influencing spatial variables between towns. With respect to processes between towns, we also have to consider the ecological flexibility of the long-legged harvestmen of the families Phalangiidae and Sclerosomatidae, which all synynthropic harvestmen from Luxembourg belong to: they all show a generalist dietary character, including vegetarian and saprobic diet in addition to predatory behaviour. This feature has been regarded a reason for the ubiquitous distribution and abundance of many Opiliones [57], [58]. Among the European species, only *O. parietinus* is strictly confined to anthropogenic habitats. The other species also thrive in semi-natural habitats. Considering the more abundant native species found in this study, *P. opilio* is a typical representative of open grasslands, while *L. rotundum*, *Oligolophus hanseni* and *Paroligolophus agrestis* inhabit light wood, forest edges and similar ecotones. Also the dominant neobiota, *O. canestrinii* and *D. ramosus* occur outside urban settlements in a variety of habitats including light forests, though in lower densities than in residential areas [29]. Urban harvestmen communities appear to be highly determined by exchange with surrounding ecosystems, thus maintaining a spatial structure across semi-natural habitats and human settlements. However, based on our results we cannot determine whether the found spatial structure refers to biotic processes that lead to a flux of individuals out of or into settlements. This interpretation of exchange among ecosystems is in line with the significant effects of semi-natural landscape structures (forests, rivers) on the synanthropic communities in Luxembourg, with landcover variables at larger scales (2000 m) being more influential, at least in the native assemblage (Table 2). Moreover, the majority of individual species show positive relationships with the proportion of agricultural and forested areas in the vicinity as well (Table 3). This urban-landscape connectivity may be favoured by the relatively small size of towns and settlements in Luxembourg. Small town size was also regarded a crucial factor in structuring urban harvestmen communities in Slovenia [22]. The studies of harvestmen suggest synanthropic communities to constitute just a mixture of species from surrounding ecosystems. This is in contrast to the results of Sattler et al. [59] who found some species to strictly occur in urban areas. These authors studied six terrestrial arthropod taxa but not harvestmen, which again suggest a possible connection to the ecology, i.e. the broad dietary niche of synanthropic harvestmen enabling them to occur in different habitats.

### Environmental variables

Even though we considered environmental variables at radii that are limited in representing microhabitat conditions, we still could explain app. 10% of the variation. This is similar to the results of Sattler et al. [20] and explained more environmental variation than other comparable studies [54], [60]. These findings demonstrate the value of considering landcover variables to explain the structure of local communities. On the other hand, the effects of the climatic parameters temperature and precipitation on community structure of synanthropic harvestmen were negligible (Table 2). The climatic differences within the county of Luxembourg may simply be too minor to show significant effects. However, also on broader scales, considering different groups of organisms across European cities, Chytrý et al. [54] did not find climatic effects. This supports the view that human alterations of habitat types have stronger impact on urban biota than large-scale climatic variables [19]. However, climatic differences may be relevant at even broader, continental scales [56].

### Variables explaining native and non-native species assemblages

The identification of processes that influence native and non-native species assemblages is a matter of high scientific interest with implications for management [9], [11], [61]. Quantifications of environmental and spatial components in different assemblage types are rare however (e.g., [10]) as are studies considering these parameters in urban areas. In synanthropic harvestmen communities of Luxembourg we found few differences in the relative contribution of environmental and spatial variables to structure native and non-native species assemblages. Although the total variation explained was somewhat higher in the non-native assemblage as compared to the native assemblage, the relative contribution of pure spatial variation was almost identical (55.8% vs 56.2% of explained variation). This finding is in contrast to the results of Sharma et al. [10], who found high levels of spatial structure in non-native fish communities that are predominantly structured by human mediated processes. In native fish communities, as much as 90% of the explained variation was governed by environmental conditions. For immigrating harvestmen, human-mediated transport appears to be of minor relevance once the species has arrived in the region. Biogeographic evidence suggests that even the most rapid harvestmen invasions in Europe follow a pattern of continuous, autonomous dispersal rather than anthropogenic long-distance dispersal [62], [63]. One exceptional case might concern *Leiobunum* spec. A [28] for which human-mediated transport is suggested to play an important role.

A further issue of crucial relevance is whether the non-native species have reached the entire study area or whether they are still in the process of expansion. Species that are expanding within the study area are thought to still be in a state of non-equilibrium with their environment, meaning that they have not yet occupied all suitable habitat [64], [65]. In this case, the expectation is that the environmental variables do not (yet) explain much of the variation in occurrence and possibly broad spatial variables explain more of it. We have little indication that this is happening in our study system, as broad, medium and fine-scaled spatial variables were selected. Additionally, the non-native harvestmen assemblage in Luxembourg is strongly dominated by a single species, *O. canestrinii*. This species occurs at high abundance throughout Luxembourg (Fig. 1) and thus probably is in state of equilibrium with its environment. We hypothesize that this fact erases differential explanatory power of environmental and spatial variables for native and non-native assemblages, which is confirmed by our results. In addition, the strong spatial overlap of native and non-native communities in the ordination plot (Fig. 3) demonstrates that species' present distributions are not principally determined by immigration history.

Despite similarities in the magnitude of spatial and environmental control of native and non-native harvestmen assemblages, there were differences in the landcover variables that explained community structure at different scales (Table 2). A positive effect of artificial urban structures was only perceptible for the non-native species, demonstrating their benefit from human facilitation (disturbance, transport, microclimate). However, the positive response to urbanization is limited even in the non-native harvestmen assemblage as shown by a hump-shaped relationship with a decline after a radius of 500 m. The initially positive effect of more available habitat by artificial structures is reversed as urbanization increases. Individual species, however, do not always follow the general trend identified for the assemblage (Table 3). The non-native species *L. religiosum*, for example, was only marginally affected by urbanization parameters. On the other hand, the native *P. opilio* showed little or negative relationship with agricultural areas and forests in the surroundings.

### Replacement of *Opilio parietinus* by *O. canestrinii* – competition-centric hypotheses reconsidered

The immigration and population increase of *O. canestrinii* coincided with the decline of *O*. *parietinus*. Among the European house wall-dwelling harvestmen, *O. parietinus* is the only fully synanthropic species, i.e. its occurrence is restricted to human settlements [22]. In the face of its dramatic population decline in many European countries [24]–[26], the species was recorded at a surprisingly high frequency in Luxembourg (at 33% of the sampled localities). It remains unclear whether this discrepancy results simply from the lack of standardized surveys on walls in the neighbouring countries, or from a true difference in population sizes. We did not find any indications of competitive replacement by *O. canestrinii*. First, a checkerboard distribution (a pattern of mutually exclusive distribution of two species) would be a particularly strong indication for competitive exclusion [66], but Fig. 1 shows a different pattern for the two *Opilio* species in Luxembourg (at 29% of all locations both species co-occur). Second, the position of the species in the ordination plot does not hint at competitive exclusion as the species stand close to each other (Fig. 4). Third, the abundance of both species was not negatively correlated (correlation coefficient R = 0.13, p = 0.33). However, one could argue that syntopic occurrence at many places is not evidence against the presence of competition. We may just witness the hot phase of competitive replacement, followed by a possible extinction of *O*. *parietinus* (“species extinction debt”, [67]). To get a reliable estimate of the population dynamics and possible interaction of the two congeners we need to include the temporal dimension. A replication via a similarly designed study in 5–10 years from now is expected to provide important insights in the presence or absence of interaction between these two model species. Of course, experimental tests of competitive effects between these species would provide the most reliable insights.

In addition, other possible causes for the decline of *O*. *parietinus* need to be considered. According to Sax et al. [9] the consequences of competition from non-native species are often overestimated. Personal observations of the microhabitats of *O. parietinus* suggest a preference for original rural structures (barns, stables, unrenovated houses), which occasionally persist even within urbanized areas. Notably, the European-wide decline of *O. parietinus* coincides with agricultural intensification. Even at the coarse scale of our landcover analysis, *O. parietinus* was the species most strongly associated with agricultural areas (Table 3). Even though agricultural area does not necessarily reflect the original rural structures mentioned above, it is still a reasonable assumption that this variable positively correlates with these structures. Interestingly, Brunzel et al. [8] found archaeophyts to occur predominantly in rural settlements. *Opilio parietinus*, the only archaeozoon among central-European Opilionids, may require similar habitat structures. Conservation strategies for *O. parietinus* therefore need to include the facilitation of structures that allow barns and stables to persist.

## Conclusions

Overall, our study points to highly dynamic arthropod communities, especially in an urban context. Synanthropic harvestmen communities in Luxembourg are largely composed of non-native species (2/3 of all specimens), a rare situation hitherto only known for some plant [9] and ant communities [68], [69]. Such a domination of non-native species is very rare among terrestrial arthropods, as we did not encounter further studies with values in a similar order of magnitude. The assemblages seem to have undergone drastic turnovers in the last decades and these changes are likely to continue due to increases in non-native species and ongoing urbanization of settlements. Native and non-native assemblages seem to be similarly structured by niche-based and neutral processes. There are no signs of competitive exclusion of native species by non-native species, instead an overall new dynamic and community structure arises. The result is a net increase in species richness, supporting the view that many communities are not saturated with species [9]. The coexistence of native and non-native species may be facilitated by the ecological flexibility of most wall-inhabiting harvestmen, giving support to the hypothesis of increased invasion success in systems of mutualistic interactions among generalists [70].

## Supporting Information

Table S1 Details of sampling localities in Luxembourg. List of sampled localities and their geographic coordinates (in decimal degrees), altitude (in meters) and number of inhabitants.
(PDF)

Table S2 Occurrence of synanthropic harvestmen at 52 localities in Luxembourg. Specimens per site matrix for each recorded harvestmen species at 52 localities across Luxembourg.
(PDF)
