# Supplementary material for: Spatial Arrangement Overrules Environmental Factors to Structure Native and Non-Native Assemblages of Synanthropic Harvestmen
Source: PLoS One. 2014 Mar 4;9(3):e90474. doi: 10.1371/journal.pone.0090474 (PMC3942446; doi:10.1371/journal.pone.0090474)
Supplement: Table S1 — Details of sampling localities in Luxembourg. List of sampled localities and their geographic coordinates (in decimal degrees), altitude (in meters) and number of inhabitants. (PDF) [file pone.0090474.s001.pdf]

Table S1. Details of sampling localities in Luxembourg.

No.	Locality	Latitude (N)	Longitude (E)	Elevation (m)	Inhabitants
1	Troisvierges	50.1214	6.0023	438	2689
2	Kalborn	50.1023	6.1121	484	54
3	Hoffelt	50.0997	5.9208	453	252
4	Clervaux	50.0537	6.0306	363	2025
5	Brachtenbach	50.0204	5.9104	475	149
6	Lellingen	49.9832	6.0131	292	86
7	Stolzembourg	49.9651	6.1673	234	170
8	Wiltz	49.965	5.9354	390	4647
9	Vianden	49.9334	6.2074	208	1782
10	Esch-sur-Sure	49.9117	5.935	286	281
11	Brandenbourg	49.9116	6.1369	284	189
12	Insenborn	49.9007	5.8836	359	157
13	Michelau	49.8992	6.0892	230	1288
14	Diekirch	49.8672	6.1596	194	6450
15	Grosbous	49.827	5.9655	352	857
16	Echternach	49.8128	6.4218	164	5100
17	Perlé	49.8103	5.7636	470	704
18	Rosport	49.8049	6.5039	160	1905
19	Pratz	49.8024	5.9378	286	347
20	Larochette	49.784	6.2194	276	1979
21	Consdorf	49.7797	6.3372	335	1759
22	Redange-sur-Attert	49.7643	5.8886	282	1288
23	Mersch	49.7506	6.1075	221	7471
24	Fischbach	49.746	6.1861	326	730
25	Moersdorf	49.7455	6.5007	147	1017
26	Saeul	49.7264	5.9863	298	550
27	Schoenfels	49.7186	6.0903	247	208
28	Eischen	49.6843	5.8782	280	1532
29	Grevenmacher	49.6782	6.4429	143	4263
30	Kopstal	49.6643	6.0725	254	3157
31	Steinfort	49.6592	5.9137	306	4324
32	Gostingen	49.6222	6.354	238	323
33	Wormeldange	49.612	6.4062	152	2450
34	Luxemburg City: Grund	49.6088	6.1361	252	781
35	Luxemburg City: Hamm	49.6042	6.17	264	1201
36	Oetrange	49.5992	6.2598	259	790
37	Hesperdange	49.5724	6.1545	273	13103
38	Syren	49.564	6.2204	278	416
39	Stadtbredimus	49.5637	6.3654	150	1500
40	Waldbredimus	49.5565	6.2865	186	954
41	Rodange	49.545	5.8393	305	5037
42	Remich-Stadt	49.5442	6.3685	144	3153
43	Bech-Kleinmacher	49.5328	6.3547	147	536
44	Wellenstein	49.5239	6.3422	180	1318
45	Differdange	49.5227	5.8883	310	20157

No.	Locality	Latitude (N)	Longitude (E)	Elevation (m)	Inhabitants
46	Lasauvage	49.5203	5.8373	301	415
47	Bettembourg	49.5162	6.103	280	9453
48	Frisange	49.5161	6.1904	247	3340
49	Schwebsange	49.5117	6.3566	152	277
50	Esch-sur-Alzette	49.4938	5.981	295	28746
51	Kayl	49.4853	6.0394	283	7788
52	Dudelange	49.4796	6.0855	291	18295
