# Supplementary material for: Spatial Arrangement Overrules Environmental Factors to Structure Native and Non-Native Assemblages of Synanthropic Harvestmen
Source: PLoS One. 2014 Mar 4;9(3):e90474. doi: 10.1371/journal.pone.0090474 (PMC3942446; doi:10.1371/journal.pone.0090474)
Supplement: Table S2 — Occurrence of synanthropic harvestmen at 52 localities in Luxembourg. Specimens per site matrix for each recorded harvestmen species at 52 localities across Luxembourg. (PDF) [file pone.0090474.s002.pdf]

Table S2. Occurrence of synanthropic harvestmen at 52 localities in Luxembourg.

Locality No.	*Amilenus aurantiacus*	*Dicranopalpus ramosus*	*Leiobunum blackwalli*	*Leiobunum religiosum*	*Leiobunum rotundum*	*Leiobunum* sp. A	*Mitopus morio*	*Odiellus spinosus*	*Oligolophus hanseni*	*Opilio canestrinii*	*Opilio parietinus*	*Opilio saxatilis*	*Paroligolophus agrestis*	*Phalangium opilio*	Individuals/Locality	Species/Locality
1										11				1	12	2
2										1					1	1
3									1	6	3			2	12	4
4					1						1				2	2
5										8		2	2		12	3
6										6	5	1			12	3
7			4		3	1	2			5				4	19	6
8										7					7	1
9					3					18	2			3	26	4
10			1		2					3				1	7	4
11										8	2			6	16	3
12										11			1	6	18	3
13					1					8				1	10	3
14		1				4				7				3	15	4
15										16	1	1		3	21	4
16		1		5		7				9	4				26	5
17					1					9	1		3	1	15	5
18		1								6				4	11	3
19					1					16		1			18	3
20	3	1	1	14	8		1		1	24			1	8	62	10
21		1								2		2	5	7	17	5
22										16				3	19	2
23		2								7				4	13	3
24					4					4			1		9	3
25		1				1				7				4	13	4
26		1			2	1				23	5			1	33	6
27			1		1	2				6				1	11	5
28				1	1	2		1	1	37		2		1	46	8
29		1			1	1		1		11				6	21	6
30					3					16				2	21	3
31		2		16	8			4		19					49	5
32											1				1	1
33										3				1	4	2
34				21	29	5				15					70	4
35			2	10	3					13			2		30	5
36		1								8				2	11	3
37		1			2					21					24	3

Locality No.	*Amilenus aurantiacus*	*Dicranopalpus ramosus*	*Leiobunum blackwalli*	*Leiobunum religiosum*	*Leiobunum rotundum*	*Leiobunum* sp. A	*Mitopus morio*	*Odiellus spinosus*	*Oligolophus hanseni*	*Opilio canestrinii*	*Opilio parietinus*	*Opilio saxatilis*	*Paroligolophus agrestis*	*Phalangium opilio*	Individuals/Locality	Species/Locality
38			1		2				2	21			1	4	31	6
39					1									1	2	2
40					2			1		12	6		1	9	31	6
41		3				1				29		2			35	4
42		2								5				11	18	3
43		1								6				18	25	3
44										6	1			17	24	3
45		1			2			1		23	2		2	2	33	7
46	1	1	1	4	5					23	5				40	7
47		3		1		1			2	12				2	21	6
48		2								11		2			15	3
49		1								6				9	16	3
50					1					9	1			1	12	4
51		6				16		2		16	1			2	43	6
52						1		1		9	1			2	14	5
Ind.	4	34	11	72	87	43	3	11	7	575	42	13	19	153	1074	
(%)	0.37	3.17	1.02	6.7	8.1	4	0.28	1.02	0.65	53.5	3.91	1.21	1.77	14.2		
Freq.	2	21	7	8	24	13	2	7	5	49	17	8	10	36		
(%)	3.85	40.4	13.5	15.4	46.2	25	3.85	13.5	9.62	94.2	32.7	15.4	19.2	69.2		
